# The Fear of SARS-CoV-2 Infection versus the Perception of COVID-19 Vaccination amongst Older Adults in Urban Areas (CoV-VAC-PL Study): A Polish Community-Based Study

**DOI:** 10.3390/vaccines12030223

**Published:** 2024-02-21

**Authors:** Mateusz Cybulski, Andrei Shpakou, Urszula Cwalina

**Affiliations:** 1 Department of Integrated Medical Care, Faculty of Health Sciences, Medical University of Bialystok, 15-096 Bialystok, Poland; andrei.shpakou@umb.edu.pl; 2 Department of Biostatistics and Medical Informatics, Faculty of Medicine with the Division of Dentistry and Division of Medical Education in English, Medical University of Bialystok, 15-295 Bialystok, Poland; urszula.cwalina@umb.edu.pl

**Keywords:** anxiety, attitudes, COVID-19, fear, older adults, SARS-CoV-2, vaccines, vaccine hesitancy

## Abstract

Background: The COVID-19 pandemic not only adversely impacted physical health but also affected older adults’ mental health. The first news on COVID-19 vaccination made a major breakthrough to the effect of improving older adults’ mood, notwithstanding the fact that vaccinated individuals in this age group accounted only for 40.6% of the overall vaccination rollout in Poland. This study was aimed at assessing the level of anxiety regarding COVID-19 amongst older adults in Poland and assessing the attitude of older adults toward COVID-19 vaccination. Methods: The study sample encompassed a population of 800 individuals aged 60 years and older randomly picked up from a representative sample of all the residents in 16 voivodeship cities (50 individuals from each of the cities). The research method used in this study was a diagnostic survey, and its technique was based on an author-designed questionnaire and four standardised psychometric scales: the Fear of COVID-19 Scale (FCV-19S), Coronavirus Anxiety Scale (CAS), the Drivers of COVID-19 Vaccination Acceptance Scale (DrVac-COVID-19S), and the Scale to Measure the Perception of SARS-CoV-2 Vaccines Acceptance (the VAC-COVID-19 Scale). Results: The degree of fear of SARS-CoV-2 among seniors equalled 1.03 ± 1.95 in terms of the CAS and 15.61 ± 5.75 in terms of the FCV-19S. Attitudes towards COVID-19 vaccination have proven positive (DrVac-COVID-19S—61.23 ± 12.35; VAC-COVID-19—44.31 ± 7.20). Females scored higher than males to the extent of the scales under consideration. The older the respondents were, the higher the scale score. A group of individuals with higher educational status was characterized by substantially higher scores covering the Knowledge subscale (*p* < 0.001) and the Autonomy subscale (*p* = 0.038), as well as a higher total score in terms of the DrVac-COVID-19S (*p* < 0.001). A group of positive factors including the reasons for COVID-19 vaccination in terms of the VAC-COVID-19 Scale was the only case to prove statistically insignificant relationships between the population size of the city the respondents came from and the scale values under consideration (*p* = 0.790). Statistically significant relationships were proven between SARS-CoV-2 contraction and fear of COVID-19 as measured by means of the CAS (*p* < 0.001) as well as between SARS-CoV-2 contraction and the Values subscale (*p* = 0.017) and the Knowledge subscale (*p* < 0.001) within the framework of the DrVac-COVID-19S scale and the total score in terms of the DrVac-COVID-19S scale (*p* = 0.023). No relationship was detected between the Autonomy subscale scores in terms of the DrVac-COVID-19S and the Knowledge subscale scores in terms of the DrVac-COVID-19S. The remaining scales were correlated to the extent of statistical significance. Conclusions: A subjective fear of COVID-19 was measured to be low or moderate within the group under study depending on the scale under consideration, proving declining trends as compared to the results arising from previously conducted studies. Seniors have more often had positive attitudes toward COVID-19 vaccination. The relationship between all of the sociodemographic features under consideration and the feeling of COVID-19 anxiety and between educational status, place of residence, SARS-CoV-2 contraction, COVID-19 vaccination, and the overall attitude toward COVID-19 vaccination indicator was proven to be statistically significant (depending on the scale under consideration). Furthermore, the correlation between the overall results arising from the standardised psychometric scales applied to this study was indicated to be statistically significant.

## 1. Introduction

The COVID-19 pandemic and related individual protection and preventive measures have accounted for major disturbances in social relations and public health care services all over the world [1]. According to the World Health Organization, on 14 January 2024 nearly 775 million COVID-19 contraction cases were confirmed and included above 7 million deaths resulting from the infection globally [2]. In Poland, according to the official statistics, 6,653,293 cases were diagnosed and included 120,458 deaths from the beginning of the pandemic until 29 January 2024 [3].

The COVID-19 pandemic not only adversely impacted the physical health of the sick but also affected the mental state of both specific groups of patients as well as the whole community [4,5]. Older adults represent a specifically vulnerable group in terms of mental health, including feelings of anxiety, strain, and depression in relation to the pandemic [6,7]. Major determinants of such a state of affairs include numerous underlying diseases (for instance, cardiovascular diseases, respiratory diseases, arterial hypertension, diabetes) and limited access to the health care system and related resources, as well as constraints to the extent of human interrelations such as social distancing and social isolation [8]. The multimorbidity phenomenon especially determines the fear of SARS-CoV-2 contraction and death amongst older adults because the higher the number of chronic diseases, the higher the risk of a severe course of COVID-19 [9]. Epidemiological data confirm that approximately ^2^/_3_ of individuals aged above 70 years suffer from at least one chronic disease [10].

The first news on COVID-19 vaccination made a major breakthrough to the effect of improving older adults’ mood. In the past, vaccines were proven to be an extremely effective way of combating epidemics [11]. Since the onset of the COVID-19 pandemic, scientists from all over the world have worked on vaccines against SARS-CoV-2 infection. Vaccines against COVID-19 have been tested and rolled out unusually fast [12,13]. The success of various vaccines against COVID-19 depends not only on their effectiveness but also on the vaccination rate of the target population, since the relevant rate of vaccination against COVID-19 will ensure protection of the vaccinated population and may constrain the spread of the virus by means of herd immunity, thus protecting even individuals who have not been vaccinated [14]. Willingness or hesitation as far vaccination is concerned is the key factor determining the vaccination coverage range. Although vaccines effectively prevent infections within the population, the COVID-19 vaccination reluctance rate continues to be high globally [15,16]. Major reasons for that include distrust of the government [17,18,19], mistrust of health care systems to the extent of standard quality vaccine assurance and vaccine side-effect management [20], vaccine safety and efficacy concerns [21,22,23,24], and low COVID-19-related personal risk that is perceived [19,22,25]. As of 29 January 2024, in Poland, 22.65 million individuals have been fully vaccinated (56.83%). During this same time, 58.46 million vaccines were administered. For the age group of 61–70 years old, 11.1 million vaccines were administered (19.0%), and for individuals aged 71–75 years, 5.5 million (9.4%), whereas for individuals aged >75 years, 7.4 million vaccines (12.7%) [26]. This means that only 41.1% of all the vaccine rollout in Poland was administered to the population of older adults. The decline in the global dynamics of the decision-making process as far as the necessity of being vaccinated is particularly worrisome.

Considering the fact that older adults constitute the group that is especially vulnerable in terms of coronaphobia (excess fear of SARS-CoV-2 infection) and severe course of COVID-19, they should account for the highest COVID-19 vaccination rate. Therefore, it is necessary to assess the level of COVID-19 anxiety and attitudes towards vaccination within the geriatric population. In Poland, representative studies have not been conducted within the group of older adults in this thematic area—the results of pilot studies conducted in this thematic area were only published at the end of the year 2022 [27].

This study was aimed at assessing the level of anxiety regarding COVID-19 amongst older adults in Poland and assessing the attitude of older adults toward COVID-19 vaccination by means of standardised psychometric scales, which will allow for comparing the results with those obtained from other international studies in this thematic area. Additionally, the relationships between a selection of sociodemographic features and the anxiety levels and attitudes toward COVID-19 vaccination amongst older adults have been evaluated altogether with the relationships between fear of SARS-CoV-2 and attitudes towards COVID-19 vaccination.

## 2. Materials and Methods

### 2.1. Participants and Study Design

The study sample encompassed a population of 800 individuals aged 60 years and older randomly picked up from a representative sample of all the residents in 16 voivodeship cities (50 individuals from each of the following cities: Białystok, Bydgoszcz, Gdańsk, Katowice, Kielce, Kraków, Lublin, Łódź, Olsztyn, Opole, Poznań, Rzeszów, Szczecin, Warszawa, Wrocław, and Zielona Góra). Random sampling was based on names from and conducted through the PESEL (Polish acronym for Universal Electronic System for Registration of the Population) Register (Ministry of Digital Affairs, Warszawa, Poland). Respective individuals were selected by means of stratified random sampling in reference to sex and age. In the course of sampling, the ultimate study efficacy was taken into account. The expected study response rate stood at RR = 25%. That made it necessary to select 3200 respondents from the cities under consideration. The following subgroups were sampled: 34 females and 34 males aged 60–69 years, 33 females and 33 males aged 70–79 years, and 33 females and 33 males aged 80 years and older (200 individuals in total) in each of the said cities.

This study was conducted by interviewers who were 2nd degree students in nursing programmes (enjoying the right to practise the nursing profession), who had been selected through the competition procedure, in the aforementioned cities during the period from 1 March to 30 November 2023. The questionnaire was implemented into the dedicated mobile application MUBSurvey 1.0 (Nfinity Sp. z o.o., Wrocław, Poland) that had been developed for the purpose of this study. Each of the interviewers obtained a login and password for their own individual user account set up by the study team, upon having installed the application on his or her own mobile device. Before the study commenced, all the interviewers had to be trained online by the study team to the extent of the application operational manual and the substance-based knowledge indispensable to acting as the interviewer; the training covered not only the instructions for filling out the questionnaire but also a practical tutorial making use of the study response scales.

Completing an informed consent form to take part in this study and a lack of cognitive disorders constituted the study inclusion criteria. The Mini Mental State Examination was performed for the purpose of screening the individuals under study in order to exclude cognitive disorders. The individuals who had scored below 27 points were ultimately excluded from the study. This gave rise to the exclusion of 17 people from the total number of 1190 individuals (1.43%). Furthermore, any respondent could withdraw at any stage of the study. Thirteen withdrawals from the study were recorded throughout the course of its duration (out of the total number of 1190 individuals), which accounted for 1.1%.

The field study was conducted in the form of personal meetings with the randomly selected respondents. It was also fully anonymised; personal particulars obtained from the Ministry of Digital Affairs, which the interviewers had been provided with, were used exclusively for organising the field study efficiently and reliably, and they were not included in the data obtained through the study. A single, full interview per one respondent took approximately 15 min on average. Should a respondent have been unable to be found and the interviewer did not receive any information on her or his whereabouts (a respondent’s death, hospitalisation, change in residence address, another reason), two subsequent attempts were made at the designated address. A respondent’s refusal to take part in the study entailed filling a reason for that in the questionnaire. In total, 360 refusals to take part in the study out of 1190 visits paid to respondents (30.25%) were recorded.

The field study conducted by the interviewers was continually supervised by the study team. In order for the interviewer to save a complete questionnaire in the application, a text message code had to be keyed in that had been received by the interviewer onto a mobile phone number previously provided. In the case of people who did not have a mobile phone, an automatic message containing a numeric code was forwarded as a voice message to the landline phone number previously provided.

### 2.2. Measures

#### 2.2.1. The Proprietary Questionnaire

The proprietary questionnaire contained questions regarding social and demographic features (sex, age, marital status, educational background, place of residence, financial standing, and professional background) as well as questions concerning SARS-CoV-2 infections in the past and a history of vaccines against COVID-19. Questions regarding the COVID-19 disease were aimed at obtaining information about whether a respondent had contracted SARS-CoV-2 in the past, confirmed by a positive test result for the real-time polymerase chain reaction (RT-PCR), and if yes, how many times she or he had become infected. In the case of questions concerning vaccinations against COVID-19, respondents were asked if they had been vaccinated, and if yes, how many vaccine doses had been administered, whether a respondent’s recovering from the disease or a symptomatic SARS-CoV-2 infection in the case of a close relative had actually influenced their decision on COVID-19 vaccination, and what sources of information on SARS-CoV-2 infections and COVID-19 vaccinations they had had access to (a multiple choice question: respondents were able to choose the following options: the Internet, television, newspapers, family, direct contacts (acquaintances, friends), medical personnel, or other persons). Questions on a subjective assessment of mental health state before the pandemic and throughout its duration (closed-ended questions with seven answer variants varied from “very bad” to “very good”) and mental disorders diagnosed by a specialist doctor in two time intervals under consideration (open-ended questions) were additionally asked.

#### 2.2.2. Fear of COVID-19 Scale (FCV-19S)

The FCV-19S is a seven-item scale developed by Ahorsu et al. [28] to measure the fear of COVID-19. The respondent can choose the following answers: “strongly disagree” (1 point), “disagree” (2 points), “neither agree nor disagree” (3 points), “agree” (4 points), and “strongly agree” (5 points). The total score was calculated by adding the score of each item (from 7 to 35). The higher the total score, the greater the feeling of fear of COVID-19 [29]. The Cronbach’s α coefficient for the Polish version of FCV-19S is α = 0.85 [30].

#### 2.2.3. Coronavirus Anxiety Scale (CAS)

The CAS is a short, self-reported scale assessing the level of fear of COVID-19. The tool consists of 5 items describing specific physical and mental ailments in response to news or thoughts about COVID-19. The respondent can choose answers from 0 points (“not at all”) to 4 points (“nearly every day over the last two weeks”). The total score is calculated by adding the score of each item (from 0 to 20). The higher the total score, the greater the feeling of fear of COVID-19 [31]. The Cronbach’s α coefficient for the Polish version of the CAS is α = 0.93 [32].

#### 2.2.4. Scale to Measure the Perception of SARS-CoV-2 Vaccines Acceptance (VAC-COVID-19)

The VAC-COVID-19 is a valid and reliable tool for measuring the perceptions of acceptance of the SARS-CoV-2 vaccine. This tool is a simple, eleven-item scale for self-administration, developed by Mejia et al. [33]. This scale distinguishes two groups of factors: positive (reasons for vaccinating) and negative (reasons for not vaccinating). Each item has been assigned five possible responses according to a Likert scale: strongly disagree (1 point), disagree (2 points), neither agree nor disagree (3 points), agree (4 points), and strongly agree (5 points). The reverse scoring applies to the second (negative) group of factors. The total score is calculated by adding the score of each item (from 11 to 55). The higher the total score, the more positive the attitude towards vaccinations against COVID-19. The Cronbach’s α coefficient for this tool is α = 0.831 [33].

#### 2.2.5. Drivers of COVID-19 Vaccination Acceptance Scale (DrVac-COVID-19S)

The DrVac-COVID-19S was adapted from the MoVac-Flu Scale [34]. The DrVac-COVID-19S contains 12 items, 9 of which being positively worded (items 1, 2, 3, 4, 5, 6, 8, 9 and 12) and 3 items being negatively worded (items 7, 10, and 11). The analysis of the tool’s results can help health care providers and researchers understand how an individual (i) cares about the goal of getting vaccinated against COVID-19 (Values) (items 3, 6, and 8); (ii) believes in the impact of vaccination against COVID-19 in order to prevent COVID-19 infection (Impacts) (items 1, 4, and 12); (iii) possesses the knowledge on vaccination against COVID-19 (Knowledge) (items 2, 5, and 10); and (iv) has the confidence and control to receive the COVID-19 vaccine if he or she wants (Autonomy) (items 7, 9, and 11). All the items are rated on a seven-point Likert scale. The reverse coding of negatively worded items (i.e., scoring them from 1 (strongly agree) to 7 (strongly disagree)) has allowed for a higher score on the DrVac-COVID-19Sto indicate a higher level of acceptance of the COVID-19 vaccine [34]. The Cronbach’s α coefficient for this tool is α = 0.912 [27].

### 2.3. Procedure and Ethical Considerations

This study was conducted after having obtained the approval of the Bioethics Committee of the Medical University of Bialystok (resolution no. APK.002.112.2023). All the subjects provided written informed consent, in accordance with the Declaration of Helsinki.

### 2.4. Statistical Analysis

The data underwent statistical analysis using the Statistica Data Miner C QC PL (StatSoft Poland, Kraków, Poland). The significance of the relationships between qualitative variables was verified using Pearson’s chi-squared test (χ^2^). The standard normal distribution of quantitative variables was checked using the Shapiro–Wilk test. Since none of the variables had normal distribution, non-parametric tests were used for analysis purposes. The significance of differences between the two groups was verified using the Mann–Whitney U test, and multiple groups were compared using the Kruskal–Wallis test and appropriate post hoc tests. Interconnections between the pairs of quantified variables were analysed using the Spearman’s rank correlation coefficients. All the test results were considered significant at *p* < 0.05.

## 3. Results

### 3.1. Sample Characteristics

In total, 800 respondents, including 401 females (50.1%) and 399 males (49.9%), took part in this study. Table 1 shows the detailed sociodemographic characteristics of the respondents, including diagnosed mental disorders and declared mental health condition before and throughout the duration of the pandemic, information on recovering from SARS-CoV-2 infection and administration of the vaccine against COVID-19, and analysis of the factors that may have an impact on vaccination decision making.

### 3.2. Descriptive Statistics of the Scales Applied to this Study

Table 2 shows descriptive statistics of the scales applied to this study. The degree of fear of SARS-CoV-2 amongst seniors has been indicated to be insignificant. The FCV-19S accounts for a higher mean degree of fear that has been kept within the moderate level. A similar case is with the attitudes towards the vaccines against COVID-19 being rather positive, which has been confirmed by the ultimate values arising from both of the scales applied to this study.

### 3.3. Impact of Sociodemographic Variables on Fear of COVID-19 and Attitudes toward COVID-19 Vaccination

Table 3 shows the relationship between sex and fear of COVID-19 and attitudes toward COVID-19 vaccination. In general, females scored higher than males in terms of the scales under consideration. The relationship between sex and the results obtained from the CAS (*p* < 0.001) and FCV-19S (*p* < 0.001) was statistically significant—males proven to feel a higher degree of fear of COVID-19 than females under this study.

Table 4 shows the relationship between age and fear of COVID-19 and attitudes toward COVID-19 vaccination. In general, the older the respondents, the higher they scored in terms of the scales under consideration. Statistically significant differences were found between age and fear of COVID-19, as measured by the CAS (*p* = 0.008) and the FCV-19S (*p* < 0.001). In the case of the FCV-19S, people aged 60–69 differed statistically significantly in this respect from people aged 70–79 (*p* = 0.009) and people aged 80 and over (*p* < 0.001). Moreover, there were statistically significant differences between age and the Values subscale (*p* < 0.001), the Impact subscale (*p* < 0.001), and the Knowledge subscale (*p* = 0.041) in terms of the DrVac-COVID-19S, including statistically significant differences in the corresponding age groups, which were also evidenced by the FCV-19S.

Table 5 shows the relationship between marital status and fear of COVID-19 and attitudes toward COVID-19 vaccination. Due to the fact that two individuals had been separated, the categories of divorced and separated were combined into one for the purpose of analysing the statistical significance. The following differences were considered statistically significant: between the widows/widowers and the married individuals (*p* = 0.027) in terms of fear of COVID-19 measured by means of the CAS, singles and widows/widowers (*p =* 0.022) and singles and the divorced/separated (*p* = 0.007) in terms of fear of COVID-19 measured by means of the FCV-19S, and singles and married individuals (*p* = 0.01) in terms of the DrVac-COVID-19S subscale assessing the effects of COVID-19 vaccination.

Table 6 shows the relationship between educational status and fear of COVID-19 and attitudes toward COVID-19 vaccination. In this case, the group of respondents with higher education scored higher. The differences proved to be statistically significant, especially as compared to the studied group with vocational training. The group of respondents with higher education was characterized by higher scores in terms of the Knowledge subscale (*p* < 0.001) and the Autonomy subscale (*p* = 0.038), as well as a higher total score in terms of the DrVac-COVID-19S (*p* < 0.001) (Table 6).

Statistically significant differences were found between professional status and fear of COVID-19 as measured by the CAS (*p* < 0.001) and the FCV-19S (*p* < 0.001). Moreover, there were statistically significant differences between professional status and the Knowledge subscale (*p* = 0.023) and the Autonomy subscale (*p* < 0.001) in terms of the DrVac-COVID-19S. Detailed data are presented in Table 7.

Table 8 shows the relationship between place of residence and fear of COVID-19 and attitudes toward COVID-19 vaccination. The group of positive factors including the reasons for COVID-19 vaccination in terms of the VAC-COVID-19 was the only one not to indicate any statistically significant relationships between the population size in the city of the respondents’ origin and the Values subscale (*p* = 0.790).

Table 9 shows the impact of recovering from SARS-CoV-2 infection on the fear of COVID-19 and attitudes towards COVID-19 vaccination. The individuals who had not recovered from SARS-CoV-2 infection proved to have a higher degree of fear of COVID-19 and adopted more negative attitudes toward COVID-19 vaccination.

Table 10 shows the relationship between COVID-19 vaccination and fear of COVID-19 and attitudes toward COVID-19 vaccination. The Autonomy subscale in terms of the DrVac-COVID-19S (*p* = 0.594) was the only one not to indicate any statistically significant relationship. The remaining results proved to be statistically significant. The detailed data are included in Table 10.

### 3.4. Correlations between Scales-Derived Values

Table 11 shows the Spearman’s rank correlations between the standardised psychometric scales applied to this study. The DrVac-COVID-19S Autonomy subscale’s values and the DrVac-COVID-19S Knowledge subscale’s values were the only ones that did not correlate. The remaining scales correlated to the extent of statistical significance.

## 4. Discussion

### 4.1. Fear of COVID-19

This study indicates that the residents in the large-sized cities in Poland aged 60 years and older show the minimum degree of fear of COVID-19 in terms of the CAS (1.03 ± 1.95) and a moderate degree in terms of the FCV-19S (15.61 ± 5.75). Our previous study indicated a substantially higher mean degree of fear of COVID-19 in terms of the CAS (6.52 ± 2.19) and a slightly higher degree in terms of the FCV-19S (17.67 ± 6.11) [27]. The results may be indicative of the fact that the health condition of Polish seniors has improved as far as fear of COVID-19 is concerned and that this social group has adapted to the current epidemiological situation.

Our study has indicated that fear of COVID-19, as measured by means of both the CAS and the FCV-19S, is significantly higher amongst females. We had similar observations within the framework of the previous study [27]. Our results are cohesive with others that have previously been published that elaborate on the differences between sex and behaviour induced by the pandemic [35,36,37,38,39]. This may be determined by the fact that males showed more health-hazardous behaviour during the COVID-19 pandemic, particularly to the extent of knowledge on the routes of SARS-CoV-2 infection. A study conducted in March–April 2020 showed that men were more reluctant to wear protective masks and abide by the social distancing policy than women [40]. Females, on the other hand, more often adapted to the then binding sanitary recommendations, which was indicative of their higher health hazard awareness as far as COVID-19 was concerned. Thereby, females may be more exposed to the strain triggering anxiety related to exposure to severe SARS-CoV-2 infection [41].

Our study has indicated that the group of the youngest individuals (60–69 years old) showed the lowest degree of fear of COVID-19 in terms of the FCV-19S. Furthermore, the differences between the youngest age group and the older age groups (70–79 years old and 80 years old and older) were proven to be statistically significant in that respect. Our previous study did not indicate any statistically significant relationship between age and fear of COVID-19 in terms of the CAS, whereas the FCV-19S accounted for a statistically significant difference [27]. The currently available related literature contains extremely diverse results. The study conducted for the Chinese population has proven that adults aged above 60 years account for the highest COVID-19-related trauma-based strain index [42], whereas other studies conducted for a variety of Chinese populations have indicated the occurrence rate of post-traumatic stress disorder [43] as well as the intensification of depression and anxiety symptoms [44] not correlated with age. Moreover, a study conducted for the Spanish population has indicated that adults aged above 65 years report less intensified depression and anxiety symptoms as compared to younger adults aged below 35 years [45]. Therefore, studies conducted in respect of the degree of fear of COVID-19 amongst seniors in relation to age require further analyses.

Our study has proven that the degree of fear of COVID-19 is higher among respondents who were widowers or had divorced. Accordingly, the study conducted by Anwar et al. [46] proved that the degree of fear of COVID-19 is higher among respondents who had lost their partners. A partner may play a key role in ensuring mental support for a counter-partner, especially in hardship such as a pandemic when access to any other support may have been limited [47,48]. In cases where one does not have a partner, these are the older adults who hardly cope with the emotions arising from an overwhelming fear of COVID-19 [49,50,51,52], which ultimately results in a higher degree of it.

In our study, we discovered that the degree of fear of COVID-19 measured by means of the CAS is indeed significantly lower among the respondents who had retired as compared to older adults who were disability/illness allowance beneficiaries. Accordingly, similar results have been obtained by Anwar et al. [46]; these researchers also discovered that the degree of fear of COVID-19 is significantly lower among the respondents who had retired. That state of affairs may be a result of poorer mental and physical health conditions and thereby a worse life quality in the case of disability/illness allowance beneficiaries.

### 4.2. Attitudes toward COVID-19 Vaccination

Our results indicated that the majority of older adults have positive attitudes toward COVID-19 vaccination. Our previous study [27] also confirmed a majority of positive attitudes toward COVID-19 vaccination, which was supported by the mean values of both of the scales DrVac-COVID-19S and VAC-COVID-19.

For over 88% of the respondents in this study, at least one vaccine dose had been administered, and for as many as 99% of them, at least two doses of the vaccine against COVID-19 had been administered. In the proprietary studies conducted in 2022 [27], the percentage share of those vaccinated equalled 88.2% of seniors, including 87.4% of those fully vaccinated (for whom at least two vaccine doses had been administered). Similar attitudes were shown by the older adults included in the study conducted by Music et al. [53]. For all the respondents in our study, at least two doses of the vaccine against COVID-19 had been administered, and almost all of them agreed that they were effective and beneficial for them and the community as a whole [53]. A study conducted in the USA among middle-aged and older adults indicated that the vast majority of their respondents support preventive vaccination, and the vaccine against COVID-19 was administered to them in May 2021 [54]. Within the framework of a Brazilian study [55] that assessed the level of acceptance of the vaccine against COVID-19 among older adults when vaccine rollout had not yet been available, 91.8% of the respondents declared their intention to be vaccinated. Similar results were obtained by American researchers in the results of a study conducted among individuals aged ≥65 years in November 2020, within the framework of which the vast majority of respondents (91%) declared acceptance of the vaccine against COVID-19 [24]. The related literature also refers to countries in which the level of acceptance of vaccination in the case of the population of older adults is significantly lower. The study conducted by Wei et al. [56] indicated that 78.3% of older adults have been vaccinated against COVID-19, and for slightly more than a half of them, a booster dose had been administered. A study conducted in Hong Kong (68.6%) indicated that 46.3% of respondents ≥55 of age were willing to be vaccinated against COVID-19 [57,58]. Accordingly, studies conducted in Saudi Arabia have indicated that out of 273 older adults aged above 60 years, 37% of them have declared their intention to be vaccinated against COVID-19 [59]. A study conducted in Russia indicated a moderate vaccine acceptance rate (50%) among older adults in their study [60].

In our study, every eighth respondent (11.7%) was not vaccinated. In the previous study, the corresponding percentage share was nearly equivalent and stood at 11.8% [27]. Almost every tenth older adult in the study conducted by Wei et al. was not interested in any vaccination [56]. According to Al-Hanawi et al. [61], over half of the older adults in Saudi Arabia (56.14%) refused to be vaccinated against COVID-19. This vaccine reluctance may partly be a result of widely disseminated misinformation on the safety and efficacy of vaccines, especially in media, including the Internet, that may evoke fear and doubts among potential vaccine recipients [62].

In our study, we found that attitudes toward COVID-19 vaccination are the most positive among the respondents aged 70–79 years, and the most negative are in the age group of 60–69 years old. In the study conducted by Ibrahim et al. [63], the COVID-19 vaccination acceptance rate was the highest among respondents aged 60–75 years and became lower the older the respondent. Related publications have proven that the COVID-19 vaccination acceptance rate among the individuals aged 75 years and older is significantly lower as compared to older adults aged 65–74 years [64]. Similar conclusions were arrived at by Wei et al. [56], as they were indicative of an explicit declining trend for the vaccination coverage rate among older adults: namely, the older the adults, the lower the vaccination coverage rate. We have not confirmed that particular observation by means of our study. The related literature highlights older adults as being characterized by a higher acceptance rate for the vaccine against COVID-19 because older adults are more exposed to SARS-CoV-2 infection [56].

Our study has indicated that older adult men show more positive attitudes towards COVID-19 vaccination than the older adult women. Ibrahim et al. [63] arrived at the same conclusions. Accordingly, a European study revealed uncertainty amongst females in all age groups as far as COVID-19 vaccination is concerned [65]. Those observations have been confirmed by other study results [66,67,68,69,70]. They may be justified by the fact that females more often express their concerns about safety and the side effects of vaccines. Related publications also include content that indicates that males account for a lower acceptance rate in respect of COVID-19 vaccination than in the case of females [71].

The educational status of the study respondents has also been an extremely important factor in determining attitudes towards COVID-19 vaccination because older adults with higher educational status have shown more positive attitudes toward vaccines, and those relationships in terms of the DrVac-COVID-19S have been statistically significant. Those results are cohesive with those arising from numerous other studies [63,69,70,72,73]. This may be corresponding with the fact that the respondents with higher education may have better access to reliable and credible information on the vaccine against COVID-19.

### 4.3. Strengths and Limitations

Our study’s strength arises from its scheme, particularly the standard normal distribution of the sample in terms of sex and age, as well as the random sampling procedure, which has allowed us to obtain representative results for the population of older adults living in the voivodeship cities in Poland. Consequently, this study exhibits important information for health care institutions and decision makers responsible for health care policy that may serve as grounds for developing the national strategy for the development of relevant COVID-19 vaccination policies addressing seniors who are uncertain about vaccines or show negative attitudes toward them.

Notwithstanding its strength as referred to above, our study also shows several limitations. Firstly, the overall results displayed arise from a study based on the subjective assessment of anxiety symptoms amongst older adults. For the purpose of this study, standardised scales were used, and although they are sensitive tools, they are based on subjective self-assessment instead of objective clinical symptoms criteria, which may result in false positive results. Secondly, this study only covered the residents in the largest cities in Poland; thereby, the overall obtained results can neither refer to residents in small-sized towns nor to older adults living in rural areas. Thirdly, geriatric patients are often characterized by multimorbidity and polypharmacy, which may intensify the fear of COVID-19, which is at the same time the motivating factor to get vaccinated against COVID-19. Other limitations of this study could have arisen from factual response bias and recall bias, which constitute common and characteristic features of any survey research.

## 5. Conclusions

The subjective degree of fear of COVID-19 in the studied group has been assessed, depending on the scale under consideration, to be low or moderate and has indicated declining trends as compared to the results arising from previous studies. Seniors have more often shown positive attitudes toward COVID-19 vaccination. The relationship between sex, age, marital status, educational status, professional status, place of residence, recovering from SARS-CoV-2 infection, COVID-19 vaccination, and fear of COVID-19 has been proven to be statistically significant. Furthermore, statistically significant relationships (depending on the scale under consideration) have been detected between educational status, place of residence, recovering from SARS-CoV-2 infection, COVID-19 vaccination, and the general attitude index in respect of the vaccine against COVID-19. The correlations between the total scores for the standardised psychometric scales applied to this study have been indicated to be statistically significant.

## Figures and Tables

**Table 1:** Respondent social and demographic characteristics.

	Feature	*n*	%
Sex	Female	401	50.1
	Male	399	49.9
Age	60–69 years old	297	37.1
	70–79 years old	252	31.5
	≥80 years old	251	31.4
Marital status	Married	479	59.9
	Separated	2	0.3
	Divorced	76	9.5
	Widow/Widower	193	24.1
Education	Higher	202	25.3
	Secondary	320	40.0
	Primary	63	7.9
	Vocational	215	26.9
Place of residence	City up to 250,000 residents	250	31.25
	City of 250,000–500,000 residents	300	37.5
	City above 500,000 residents	250	31.25
Financial situation	Very bad	10	1.3
	Bad	0	0.0
	Rather bad	16	2.0
	Neither good nor bad	141	17.6
	Rather good	146	18.3
	Good	378	47.3
	Very good	109	13.6
Professional status	I have retired	647	80.9
	I am a disability/illness allowance beneficiary	29	3.6
	I am professionally active (I work)	124	15.5
Health condition self-assessment before the pandemic	Very bad	1	0.1
	Bad	1	0.1
	Rather bad	13	1.6
	Neither good nor bad	54	6.8
	Rather good	153	19.1
	Good	381	47.6
	Very good	19	24.6
Mental disorders before the pandemic	Yes	35	4.4
	No	765	95.6
Mental disorders diagnosed before the pandemic	Depression	17	2.1
	Panic disorder	1	0.1
	Neurosis	1	0.1
	Sleep disorders	16	2.1
Health condition self-assessment during the pandemic	Very bad	0	0.0
	Bad	8	1.0
	Rather bad	22	2.8
	Neither good nor bad	93	11.6
	Rather good	196	24.5
	Good	322	40.3
	Very good	159	19.9
Mental disorders during the pandemic	Yes	4	0.5
	No	796	99.5
Mental disorders diagnosed during the pandemic	Depression	1	0.1
	Panic disorder	1	0.1
	Agoraphobia	1	0.1
	Sleep disorders	1	0.1
SARS-CoV-2 infection confirmed by a PCR test positive result	Yes	304	38.0
	No	496	62.0
Number of SARS-CoV-2 infections	1	204	67.1
	2	78	25.7
	3	20	6.6
	4	2	0.7
COVID-19 vaccination	Yes	706	88.3
	No	94	11.7
Number of vaccine doses against COVID-19	1	11	1.6
	2	168	23.8
	3	350	49.6
	4	164	23.2
	5	13	1.8
Sources of information on COVID-19 and vaccines against COVID-19 ^(1)^	Internet	295	36.9
	Television	643	80.4
	Press	371	46.4
	Family	457	57.1
	Acquaintances, friends	361	45.1
	Medical personnel	312	39.0
The vaccination decision was influenced by my recovering from the disease or a symptomatic SARS-CoV-2 infection in the case of a close relative.	Yes	295	36.9
	No	505	63.1
What role did your family and direct contacts (acquaintances, friends) play as far as COVID-19 vaccination was concerned?	They encouraged me to be vaccinated against COVID-19.	344	43.0
	They encouraged me to be vaccinated against COVID-19.	177	22.1
	Difficult to say.	279	34.9

^(1)^—The total score does not have to equal 100% since one may choose any number of answers.

**Table 2:** Descriptive statistics of scales applied to this study.

Scale	M	SD	Min	Q_25_	Me	Q_75_	Max
CAS	1.03	1.95	0.0	0.0	0.0	1.0	13.0
FCV-19S	15.61	5.75	7.0	11.0	16.0	19.0	35.0
Values subscale	15.24	4.69	3.0	13.0	16.5	18.0	21.0
Impacts subscale	15.63	4.48	3.0	14.0	17.0	19.0	21.0
Knowledge subscale	13.15	3.95	3.0	10.0	14.0	16.0	21.0
Autonomy subscale	17.21	2.94	8.0	15.0	18.0	19.0	21.0
DrVac-COVID-19S-total	61.23	12.35	25.0	54.0	64.0	70.0	84.0
VAC-COVID-19-negative subscale	28.33	4.86	7.0	26.0	29.0	32.0	35.0
VAC-COVID-19-positive subscale	15.98	3.23	4.0	14.0	16.0	18.0	20.0
VAC-COVID-19-total	44.31	7.20	18.0	41.0	45.0	50.0	55.0

Abbreviations: CAS—Coronavirus Anxiety Scale; DrVac-COVID-19S—Drivers of COVID-19 Vaccination Acceptance Scale; FCV-19S—Fear of COVID-19 Scale; M—mean; Max—maximum; Me—median; Min—minimum; SD—standard deviation; Q_25_—lower quartile; Q_75_—upper quartile; VAC-COVID-19—Scale to Measure the Perception of SARS-CoV-2 Vaccines Acceptance.

**Table 3:** Relationship between sex and fear of COVID-19 and attitudes toward COVID-19 vaccination.

Scale	Women (*n* = 401)		Men (*n* = 399)		*p* ^a^
	M ± SD	Me (IQR)	M ± SD	Me (IQR)	
CAS	1.18 ± 1.98	0 (0–2)	0.87 ± 1.90	0 (0–1)	<0.001 *
FCV-19S	16.38 ± 5.72	16 (13–20)	14.83 ± 5.67	15 (10–18)	<0.001 *
Values subscale	15.42 ± 4.55	17 (13–18)	15.05 ± 4.83	16 (13–18)	0.324
Impacts subscale	15.62 ± 4.47	17 (14–19)	15.64 ± 4.49	17 (14–18)	0.877
Knowledge subscale	13.30 ± 4.03	14 (10–16)	13.00 ± 3.87	13 (10–16)	0.235
Autonomy subscale	17.25 ± 2.90	18 (16–19)	17.17 ± 2.98	18 (15–19)	0.785
DrVac-COVID-19S	61.60 ± 12.17	64 (54–71)	60.85 ± 12.53	64 (54–70)	0.392
VAC-COVID-19-negative subscale	28.44 ± 4.92	29 (26–32)	28.21 ± 4.80	28 (26–32)	0.350
VAC-COVID-19-positive subscale	16.07 ± 3.26	16 (14–18)	15.89 ± 3.20	16 (14–18)	0.360
VAC-COVID-19-total score	44.51 ± 7.28	46 (40–50)	44.10 ± 7.13	45 (41–50)	0.318

Abbreviations: ^a^—Mann–Whitney U test; CAS—Coronavirus Anxiety Scale; DrVac-COVID-19S—Drivers of COVID-19 Vaccination Acceptance Scale; FCV-19S—Fear of COVID-19 Scale; M—mean; Me—median; IQR—interquartile range; *p*—*p*-value; SD—standard deviation; VAC-COVID-19—Scale to Measure the Perception of SARS-CoV-2 Vaccines Acceptance; *—statistically significant.

**Table 4:** Relationship between age and fear of COVID-19 and attitudes toward COVID-19 vaccination.

Scale	60–69 Years (I) (*n* = 297)		70–79 Years (II) (*n* = 252)		80 and More Years (III) (*n* = 251)		*p* ^a^	*p* ^b^
	M ± SD	Me (IQR)	M ± SD	Me (IQR)	M ± SD	Me (IQR)		
CAS	0.74 ± 1.67	0 (0–1)	1.13 ± 1.96	0 (0–1)	1.27 ± 2.18	0 (0–2)	0.008 *	I-II: 0.096 I-III: 0.061 II-III: 1
FCV-19S	14.63 ± 6.05	14 (9–18)	15.81 ± 5.34	16 (13–18)	16.55 ± 5.61	16 (12–20)	<0.001 *	I-II: 0.009 * I-III: <0.001 * II-III: 0.542
Values subscale	14.45 ± 4.88	16 (12–18)	15.56 ± 4.69	17 (14–18)	15.84 ± 4.35	17 (14–19)	<0.001 *	I-II: 0.01 * I-III: 0.001 * II-III: 1
Impacts subscale	14.86 ± 4.73	16 (13–18)	15.92 ± 4.44	17 (14.5–19)	16.26 ± 4.08	18 (14–19)	<0.001 *	I-II: 0.015 * I-III: <0.001 * II-III: 1
Knowledge subscale	13.41 ± 3.91	14 (10–16)	13.36 ± 3.93	14 (10–16)	12.63 ± 3.99	13 (10–16)	0.041 *	I-II: 1 I-III: 0.074 II-III: 0.097
Autonomy subscale	17.33 ± 2.88	18 (16–19)	17.29 ± 2.84	18 (15.5–19)	16.98 ± 3.12	18 (15–19)	0.431	-
DrVac-COVID-19S	60.05 ± 12.87	63 (51–70)	62.13 ± 12.15	66 (56–70)	61.71 ± 11.84	64 (55–70)	0.121	-
VAC-COVID-19-negative subscale	28.42 ± 5.09	29 (26–32)	28.33 ± 4.58	29 (26–32)	28.20 ± 4.87	28 (25–32)	0.637	-
VAC-COVID-19-positive subscale	15.58 ± 3.54	16 (14–18)	16.27 ± 2.97	16 (14–18)	16.17 ± 3.05	16 (15–18)	0.112	-
VAC-COVID-19-total score	44.01 ± 7.71	45 (40–50)	44.60 ± 6.77	46 (41–50)	44.37 ± 7.03	45 (41–50)	0.869	-

Abbreviations: ^a^—Kruskal–Wallis test; ^b^—post hoc test; CAS—Coronavirus Anxiety Scale; DrVac-COVID-19S—Drivers of COVID-19 Vaccination Acceptance Scale; FCV-19S—Fear of COVID-19 Scale; M—mean; Me—median; IQR—interquartile range; *p*—*p*-value; SD—standard deviation; VAC-COVID-19—Scale to Measure the Perception of SARS-CoV-2 Vaccines Acceptance; *—statistically significant.

**Table 5:** Relationship between marital status and fear of COVID-19 and attitudes toward COVID-19 vaccination.

Scale	Single (I) (*n* = 50)		Widow/Widower (II) (*n* = 193)		Married (III) (*n* = 479)		Divorced/Separated (IV) (*n* = 78)		*p* ^a^	*p* ^b^
	M ± SD	Me (IQR)	M ± SD	Me (IQR)	M ± SD	Me (IQR)	M ± SD	Me (IQR)		
CAS	0.88 ± 2.06	0 (0–0)	1.31 ± 2.01	0 (0–2)	0.89 ± 1.76	0 (0–1)	1.32 ± 2.64	0 (0–1)	0.002 *	I-II: 0.237 I-III: 1 I-IV: 0.845 II-III: 0.027 * II-IV: 1 III-IV: 0.820
FCV-19S	13.84 ± 6.50	12.5 (8–18)	16.25 ± 5.74	16 (12–20)	15.30 ± 5.58	15 (11–19)	17.04 ± 5.90	17 (14–19)	0.003 *	I-II: 0.022 * I-III: 0.236 I-IV: 0.007 * II-III: 0.411 II-IV: 1 III-IV: 0.126
Values subscale	12.98 ± 6.36	15 (6–18)	15.42 ± 4.44	17 (13–18)	15.41 ± 4.57	17 (13–18)	15.15 ± 4.54	16 (13–18)	0.103	-
Impacts subscale	13.12 ± 6.07	15 (7–18)	15.54 ± 4.46	17 (13–18)	15.94 ± 4.25	17 (14–19)	15.54 ± 4.24	17 (14–18)	0.016 *	I-II: 0.094 I-III: 0.01 * I-IV: 0.328 II-III: 1 II-IV: 1 III-IV: 1
Knowledge subscale	13.24 ± 4.28	14 (11–16)	12.93 ± 4.04	13 (10–16)	13.38 ± 3.84	14 (10–16)	12.26 ± 4.12	12,5 (9–15)	0.133	-
Autonomy subscale	16.76 ± 3.01	17 (15–19)	16.97 ± 3.11	18 (15–19)	17.22 ± 2.94	18 (15–19)	18.04 ± 2.30	18 (17–19)	0.043 *	I-II: 1 I-III: 1 I-IV: 0.085 II-III: 1 II-IV: 0.083 III-IV: 0.237
DrVac-COVID-19S	56.10 ± 15.95	59 (42–68)	60.87 ± 12.38	64 (53–70)	61.95 ± 11.83	65 (54–70)	60.99 ± 12.17	63 (54–70)	0.087	-
VAC-COVID-19-negative subscale	27.02 ± 5.69	28.5 (22–32)	28.01 ± 4.87	28 (25–32)	28.63 ± 4.83	29 (26–32)	28.08 ± 4.30	28 (26–31)	0.100	-
VAC-COVID-19-positive subscale	15.10 ± 4.00	16 (12–18)	16.13 ± 3.10	16 (14–18)	16.04 ± 3.21	16 (14–18)	15.81 ± 3.09	16 (13–18)	0.442	-
VAC-COVID-19-total score	42.12 ± 8.67	46 (36–49)	44.14 ± 7.21	45 (40–50)	44.67 ± 7.10	45 (41–50)	43.88 ± 6.66	44 (40–49)	0.187	-

Abbreviations: ^a^—Kruskal–Wallis test; ^b^—post hoc test; CAS—Coronavirus Anxiety Scale; DrVac-COVID-19S—Drivers of COVID-19 Vaccination Acceptance Scale; FCV-19S—Fear of COVID-19 Scale; M—mean; Me—median; IQR—interquartile range; *p*—*p*-value; SD—standard deviation; VAC-COVID-19—Scale to Measure the Perception of SARS-CoV-2 Vaccines Acceptance; *—statistically significant.

**Table 6:** Relationship between educational status and fear of COVID-19 and attitudes toward COVID-19 vaccination.

Scale	Primary (I) (*n* = 63)		Secondary (II) (*n* = 320)		Vocational (III) (*n* = 215)		Higher (IV) (*n* = 202)		*p* ^a^	*p* ^b^
	M ± SD	Me (IQR)	M ± SD	Me (IQR)	M ± SD	Me (IQR)	M ± SD	Me (IQR)		
CAS	0.97 ± 1.75	0 (0–1)	1.11 ± 2.01	0 (0–1)	1.19 ± 1.98	0 (0–2)	0.75 ± 1.84	0 (0–1)	0.01 *	I-II: 1 I-III: 1 I-IV: 1 II-III: 1 II-IV: 0.178 III-IV: 0.038 *
FCV-19S	16.21 ± 5.72	16 (12–20)	15.57 ± 5.74	16 (11–19)	16.45 ± 5.96	17 (13–20)	14.58 ± 5.40	14 (10–18)	0.002 *	I-II: 1 I-III: 1 I-IV: 0.1780 II-III: 0.269 II-IV: 0.258 III-IV: 0.001 *
Values subscale	14.40 ± 5.10	16 (12–18)	15.52 ± 4.45	17 (14–18)	14.69 ± 4.95	16 (13–18)	15.63 ± 4.60	17 (13–19)	0.068	-
Impacts subscale	15.05 ± 4.59	17 (12–18)	15.81 ± 4.32	17 (14–18.5)	15.11 ± 4.74	16 (14–18)	16.08 ± 4.37	17 (14–19)	0.065	-
Knowledge subscale	11.29 ± 3.76	11 (9–14)	13.11 ± 4.01	14 (10–16)	12.47 ± 3.68	12 (10–15)	14.52 ± 3.78	15 (12–17)	<0.001 *	I-II: 0.003 * I-III: 0.236 I-IV: <0.001 * II-III: 0.244 II-IV: <0.001 * III-IV: <0.001 *
Autonomy subscale	17.06 ± 2.76	18 (15–19)	17.20 ± 3.07	18 (16–19)	16.91 ± 2.78	18 (15–19)	17.59 ± 2.94	18 (16–20)	0.046 *	I-II: 1 I-III: 1 I-IV: 0.827 II-III: 0.711 II-IV: 0.896 III-IV: 0.038 *
DrVac-COVID-19S	57.79 ± 11.86	60 (49–67)	61.64 ± 11.63	64 (54–70)	59.17 ± 12.80	61 (53–69)	63.83 ± 12.58	66 (57–73)	<0.001 *	I-II: 0.147 I-III: 1 I-IV: <0.001 * II-III: 0.404 II-IV: 0.056 III-IV: <0.001 *
VAC-COVID-19-negative subscale	27.11 ± 6.31	29 (23–32)	28.52 ± 4.64	29 (26–32)	27.58 ± 4.90	28 (25–32)	29.20 ± 4.46	30 (26–33)	0.006 *	I-II: 1 I-III: 1 I-IV: 0.240 II-III: 0.192 II-IV: 0.761 III-IV: 0.005 *
VAC-COVID-19-positive subscale	15.97 ± 3.84	17 (13–19)	15.99 ± 3.25	16 (14–18)	15.85 ± 3.12	16 (14–18)	16.12 ± 3.13	16 (14–18)	0.720	-
VAC-COVID-19-total score	43.08 ± 9.32	47 (36–51)	44.50 ± 7.03	45 (41–50)	43.42 ± 7.20	44 (40–49)	45.32 ± 6.60	46 (41–51)	0.072	-

Abbreviations: ^a^—Kruskal–Wallis test; ^b^—post hoc test; CAS—Coronavirus Anxiety Scale; DrVac-COVID-19S—Drivers of COVID-19 Vaccination Acceptance Scale; FCV-19S—Fear of COVID-19 Scale; M—mean; Me—median; IQR—interquartile range; *p*—*p*-value; SD—standard deviation; VAC-COVID-19—Scale to Measure the Perception of SARS-CoV-2 Vaccines Acceptance; *—statistically significant.

**Table 7:** Relationship between professional status and fear of COVID-19 and attitudes toward COVID-19 vaccination.

Scale	Disability/Illness Allowance (I) (*n* = 29)		Pension (II) (*n* = 647)		Professionally Active (III) (*n* = 124)		*p* ^a^	*p* ^b^
	M ± SD	Me (IQR)	M ± SD	Me (IQR)	M ± SD	Me (IQR)		
CAS	3.31 ± 2.84	4 (0–6)	0.98 ± 1.85	0 (0–1)	0.74 ± 1.83	0 (0–1)	<0.001 *	I-II: <0.001 * I-III: <0.001 * II-III: 0.708
FCV-19S	18.07 ± 4.80	19 (17–20)	15.79 ± 5.67	16 (12–19)	14.08 ± 6.03	13.5 (9–17.5)	<0.001 *	I-II: 0.016 I-III: <0.001 * II-III: <0.001*
Values subscale	15.17 ± 3.05	15 (14–16)	15.33 ± 4.64	17 (13–18)	14.74 ± 5.24	17 (11.5–18)	0.239	-
Impacts subscale	15.34 ± 2.68	16 (14–16)	15.76 ± 4.42	17 (14–19)	15.03 ± 5.06	16.5 (12.5–18)	0.098	-
Knowledge subscale	14.38 ± 3.00	15 (13–16)	12.98 ± 3.95	13 (10–16)	13.78 ± 4.08	14 (10.5–17)	0.023 *	I-II: 0.162 I-III: 1 II-III: 0.111
Autonomy subscale	13.38 ± 3.57	12 (11–16)	17.28 ± 2.80	18 (16–19)	17.72 ± 2.90	18 (16.5–20)	<0.001 *	I-II: <0.001 * I-III: <0.001 * II-III: 0.202
DrVac-COVID-19S	58.28 ± 8.20	57 (54–60)	61.35 ± 12.17	64 (54–70)	61.27 ± 13.94	65 (51–71)	0.072	-
VAC-COVID-19-negative subscale	28.28 ± 3.32	28 (27–31)	28.22 ± 4.89	28 (25–32)	28.87 ± 4.97	30 (27–32)	0.210	-
VAC-COVID-19-positive subscale	16.00 ± 2.63	16 (16–17)	16.04 ± 3.19	16 (14–18)	15.68 ± 3.53	16 (14–18)	0.694	-
VAC-COVID-19-total score	44.28 ± 5.18	44 (43–47)	44.26 ± 7.22	45 (40–50)	44.55 ± 7.59	46 (41.5–50)	0.672	-

Abbreviations: ^a^—Kruskal–Wallis test; ^b^—post hoc test; CAS—Coronavirus Anxiety Scale; DrVac-COVID-19S—Drivers of COVID-19 Vaccination Acceptance Scale; FCV-19S—Fear of COVID-19 Scale; M—mean; Me—median; IQR—interquartile range; *p*—*p*-value; SD—standard deviation; VAC-COVID-19—Scale to Measure the Perception of SARS-CoV-2 Vaccines Acceptance; *—statistically significant.

**Table 8:** Relationship between place of residence and fear of COVID-19 and attitudes toward COVID-19 vaccination.

Scale	City of up to 250,000 Residents (I) (*n* = 250)		City of 250,000–500,000 Residents (II) (*n* = 300)		City Above 500,000 Residents (III) (*n* = 250)		*p* ^a^	*p* ^b^
	M ± SD	Me (IQR)	M ± SD	Me (IQR)	M ± SD	Me (IQR)		
CAS	1.73 ± 2.12	1 (0–3)	0.76 ± 1.88	0 (0–0)	0.66 ± 1.64	0 (0–0)	<0.001 *	I-II: <0.001 * I-III: <0.001 * II-III: 1
FCV-19S	16.40 ± 3.96	17 (14–19)	16.37 ± 6.26	16 (12–19)	13.90 ± 6.24	12 (9–18)	<0.001 *	I-II: 0.260 I-III: <0.001 * II-III: <0.001 *
Values subscale	16.36 ± 3.57	17 (15–18)	15.01 ± 4.30	16 (12–18)	14.38 ± 5.81	16 (10–19)	<0.001 *	I-II: 0.002 * I-III: 0.008 * II-III: 1
Impacts subscale	16.50 ± 3.42	17 (15–18)	15.68 ± 4.04	16.5 (14–18)	14.70 ± 5.61	16 (11–19)	0.021 *	I-II: 0.110 I-III: 0.025 * II-III: 1
Knowledge subscale	13.89 ± 3.57	15 (12–16)	12.96 ± 3.82	13 (10–16)	12.64 ± 4.36	13 (9–16)	<0.001 *	I-II: 0.008 * I-III: <0.001 * II-III: 1
Autonomy subscale	16.50 ± 3.13	18 (14–19)	17.19 ± 2.65	18 (16–19)	17.94 ± 2.91	18 (16–21)	<0.001 *	I-II: 0.242 I-III: <0.001 * II-III: <0.001*
DrVac-COVID-19S	63.26 ± 10.23	65 (58–70)	60.84 ± 11.61	62 (54–70)	59.66 ± 14.69	63 (48–71)	0.026 *	I-II: 0.063 I-III: 0.049 * II-III: 1
VAC-COVID-19-negative subscale	29.64 ± 4.22	30 (27–33)	27.07 ± 4.86	27 (24–31)	28.52 ± 5.10	30 (26–32)	<0.001 *	I-II: <0.001 * I-III: 0.097 II-III: <0.001 *
VAC-COVID-19-positive subscale	16.29 ± 2.34	16 (15–18)	16.00 ± 3.10	16 (14–19)	15.66 ± 4.03	17 (13–19)	0.790	-
VAC-COVID-19-total score	45.93 ± 5.87	47 (43–50)	43.07 ± 7.00	43 (39–48)	44.18 ± 8.29	47 (39–50)	<0.001 *	I-II: <0.001 * I-III: 0.318 II-III: 0.004 *

Abbreviations: ^a^—Kruskal–Wallis test; ^b^—post hoc test; CAS—Coronavirus Anxiety Scale; DrVac-COVID-19S—Drivers of COVID-19 Vaccination Acceptance Scale; FCV-19S—Fear of COVID-19 Scale; M—mean; Me—median; IQR—interquartile range; *p*—*p*-value; SD—standard deviation; VAC-COVID-19—Scale to Measure the Perception of SARS-CoV-2 Vaccines Acceptance; *—statistically significant.

**Table 9:** Relationship between the impact of SARS-CoV-2 infection on fear of COVID-19 and attitudes towards COVID-19 vaccination.

Scale	SARS-CoV-2 Infection (*n* = 304)		No SARS-CoV-2 Infection (*n* = 496)		*p* ^a^
	M ± SD	Me (IQR)	M ± SD	Me (IQR)	
CAS	1.49 ± 2.38	0 (0–2)	0.75 ± 1.56	0 (0–1)	<0.001 *
FCV-19S	16.01 ± 5.70	16 (12–19)	15.36 ± 5.77	15 (11–19)	0.096
Values subscale	15.84 ± 4.21	17 (14–18)	14.86 ± 4.93	16 (12–18)	0.017 *
Impacts subscale	15.99 ± 4.09	17 (14–18.5)	15.41 ± 4.69	17 (13–19)	0.212
Knowledge subscale	13.71 ± 3.76	14 (11–16)	12.81 ± 4.03	13 (10–16)	0.001 *
Autonomy subscale	17.03 ± 3.15	18 (15–19)	17.32 ± 2.81	18 (16–19)	0.400
DrVac-COVID-19S	62.58 ± 11.63	65 (56–70)	60.40 ± 12.71	63 (52.5–70)	0.023 *
VAC-COVID-19-negative subscale	28.55 ± 4.49	29 (26–32)	28.19 ± 5.07	29 (26–32)	0.600
VAC-COVID-19-positive subscale	16.09 ± 3.13	16 (14.5–18)	15.92 ± 3.29	16 (14–18)	0.599
VAC-COVID-19-total score	44.64 ± 6.68	45 (41–50)	44.10 ± 7.51	45 (40–50)	0.625

Abbreviations: ^a^—Mann–Whitney U test; CAS—Coronavirus Anxiety Scale; DrVac-COVID-19S—Drivers of COVID-19 Vaccination Acceptance Scale; FCV-19S—Fear of COVID-19 Scale; M—mean; Me—median; IQR—interquartile range; *p*—*p*-value; SD—standard deviation; VAC-COVID-19—Scale to Measure the Perception of SARS-CoV-2 Vaccines Acceptance; *—statistically significant.

**Table 10:** Relationship between COVID-19 vaccination and fear of COVID-19 and attitudes toward COVID-19 vaccination.

Scale	COVID-19 Vaccination (*n* = 706)		No COVID-19 Vaccination (*n* = 94)		*p* ^a^
	M ± SD	Me (IQR)	M ± SD	Me (IQR)	
CAS	1.13 ± 2.00	0 (0–2)	0.30 ± 1.29	0 (0–0)	<0.001 *
FCV-19S	16.12 ± 5.58	16 (12–19)	1.74 ± 5.56	10.5 (7–15)	<0.001 *
Values subscale	16.36 ± 3.53	17 (15–19)	6.78 ± 3.57	6 (4–9)	<0.001 *
Impacts subscale	16.66 ± 3.45	17 (15–19)	7.90 ± 3.73	8 (5–11)	<0.001 *
Knowledge subscale	13.62 ± 3.77	14 (11–16)	9.60 ± 3.42	9 (7–11)	<0.001 *
Autonomy subscale	17.17 ± 2.99	18 (15–19)	17.52 ± 2.51	18 (16–19)	0.594
DrVac-COVID-19S	63.81 ± 10.28	65.5 (58–71)	41.80 ± 8.76	41 (36–48)	<0.001 *
VAC-COVID-19-negative subscale	29.21 ± 4.14	30 (27–33)	21.68 ± 4.71	21 (19–24)	<0.001 *
VAC-COVID-19-positive subscale	16.62 ± 2.62	17 (15–19)	11.20 ± 3.36	12 (9–13)	<0.001 *
VAC-COVID-19-total score	45.83 ± 5.80	46 (42–50)	32.88 ± 6.44	32 (28–36)	<0.001 *

Abbreviations: ^a^—Mann–Whitney U test; CAS—Coronavirus Anxiety Scale; DrVac-COVID-19S—Drivers of COVID-19 Vaccination Acceptance Scale; FCV-19S—Fear of COVID-19 Scale; M—mean; Me—median; IQR—interquartile range; *p*—*p*-value; SD—standard deviation; VAC-COVID-19—Scale to Measure the Perception of SARS-CoV-2 Vaccines Acceptance; *—statistically significant.

**Table 11:** Spearman’s rank correlation between standardised psychometric scales applied to this study.

Scale		CAS	FCV-19S	V	I	K	A	DrVac- COVID-19S	VAC- COVID-19-Negative	VAC- COVID-19-Positive
FCV-19S	r	0.550								
	*p*	<0.001 *								
Values (V) subscale	r	0.196	0.204							
	*p*	<0.001 *	<0.001 *							
Impacts (I) subscale	r	0.163	0.198	0.885						
	*p*	<0.001 *	<0.001 *	<0.001 *						
Knowledge (K) subscale	r	0.114	0.134	0.520	0.520					
	*p*	<0.001 *	<0.001 *	<0.001 *	<0.001 *					
Autonomy (A) subscale	r	−0.211	−0.237	0.230	0.258	0.004				
	*p*	<0.001 *	<0.001 *	<0.001 *	<0.001 *	0.917				
DrVac-COVID-19S	r	0.114	0.161	0.899	0.906	0.715	0.390			
	*p*	<0.001 *	<0.001 *	<0.001 *	<0.001 *	<0.001 *	<0.001 *			
VAC-COVID-19-negative subscale	r	0.159	0.152	0.557	0.517	0.408	0.215	0.573		
	*p*	<0.001 *	<0.001 *	<0.001 *	<0.001 *	<0.001 *	<0.001 *	<0.001 *		
VAC-COVID-19-positive subscale	r	0.120	0.223	0.554	0.545	0.349	0.145	0.566	0.563	
	*p*	<0.001 *	<0.001 *	<0.001 *	<0.001 *	<0.001 *	<0.001 *	<0.001 *	<0.001 *	
VAC-COVID-19-total score	r	0.159	0.206	0.612	0.579	0.428	0.203	0.629	0.926	0.820
	*p*	<0.001 *	<0.001 *	<0.001 *	<0.001 *	<0.001 *	<0.001 *	<0.001 *	<0.001 *	<0.001 *

Abbreviations: A—DrVac-COVID-19S Autonomy subscale; CAS—Coronavirus Anxiety Scale; DrVac-COVID-19S—Drivers of COVID-19 Vaccination Acceptance Scale; FCV-19S—Fear of COVID-19 Scale; I—DrVac-COVID-19S Impacts subscale; K—DrVac-COVID-19S Knowledge subscale; *p*—*p*-value; r—Spearman’s rank correlation coefficient; V—DrVac-COVID-19S Values subscale; VAC-COVID-19—Scale to Measure the Perception of SARS-CoV-2 Vaccines Acceptance; *—statistically significant.

## Data Availability

The data presented in this study are available on request from the corresponding author. The data are not publicly available due to information that could compromise the privacy of research participants.
